# Tissue, age, sex, and disease patterns of matrisome expression in GTEx transcriptome data

**DOI:** 10.1038/s41598-021-00943-x

**Published:** 2021-11-03

**Authors:** Tim O. Nieuwenhuis, Avi Z. Rosenberg, Matthew N. McCall, Marc K. Halushka

**Affiliations:** 1 Department of Pathology, Johns Hopkins University School of Medicine, Baltimore, MD 21205 USA; 2 Department of Biostatistics and Computational Biology, University of Rochester Medical Center, Rochester, NY 14642 USA

**Keywords:** Systems biology, Functional genomics, Gene expression, Genomics, Proteomics

## Abstract

The extracellular matrix (ECM) has historically been explored through proteomic methods. Whether or not global transcriptomics can yield meaningful information on the human matrisome is unknown. Gene expression data from 17,382 samples across 52 tissues, were obtained from the Genotype-Tissue Expression (GTEx) project. Additional datasets were obtained from The Cancer Genome Atlas (TCGA) program and the Gene Expression Omnibus for comparisons. Gene expression levels generally matched proteome-derived matrisome expression patterns. Further, matrisome gene expression properly clustered tissue types, with some matrisome genes including SERPIN family members having tissue-restricted expression patterns. Deeper analyses revealed 382 gene transcripts varied by age and 315 varied by sex in at least one tissue, with expression correlating with digitally imaged histologic tissue features. A comparison of TCGA tumor, TCGA adjacent normal and GTEx normal tissues demonstrated robustness of the GTEx samples as a generalized matrix control, while also determining a common primary tumor matrisome. Additionally, GTEx tissues served as a useful non-diseased control in a separate study of idiopathic pulmonary fibrosis (IPF) matrix changes, while identifying 22 matrix genes upregulated in IPF. Altogether, these findings indicate that the transcriptome, in general, and GTEx in particular, has value in understanding the state of organ ECM.

## Introduction

The extracellular matrix (ECM) is the non-cellular scaffold found across tissues that provides structural integrity and mediates signaling to the cells with which it interacts^1,2^. The ECM is generated primarily by mesenchymal and other surrounding cells (fibroblasts, epithelial cells, adipocytes, etc.) during development and throughout the lifespan of an organism. It is primarily comprised of proteins, polysaccharides, and water, the exact type and proportions of each being unique to each tissue. This tissue specificity of the ECM is vital to the structure and function of each organ, with mutations of ECM genes resulting in Mendelian diseases that variably affects organs^3,4^.

While the study of ECM components predates cellular theory^5^, the cataloging of its protein components has been a long endeavor. Due to the biochemical characteristics of ECM, such as its insolubility and frequent crosslinking, its composition has been difficult to interrogate even with the advancement of proteomic techniques^6^. The Hynes group catalogued a so-called “matrisome” using a combination of proteomic techniques applied to decellularized tissue samples and in silico bioinformatics analyses^7^. Their categorization is separated into two primary divisions, the core-matrisome proteins (n = 274) and matrisome-associated proteins (n = 753). The core-matrisome consists of three categories: collagens, proteoglycans, and ECM glycoproteins. These represent the structural elements of the ECM. The matrisome-associated protein category, contrastingly, includes ECM regulators, ECM-affiliated proteins, and secreted factors.

While the majority of research in the ECM has been performed through mass spectrometry and immunohistochemistry, the field has not been comprehensively interrogated using transcriptomics. Herein, we curate a tissue-wide transcriptomic landscape of the core-matrisome and ECM regulators in normal human samples, and further investigate how the matrisome transcriptome is perturbed in lung disease and cancer. We use GTEx’s bulk sequencing data of 54 tissues comprised of 17,382 samples to create a generalized understanding of the similarities and differences of ECM expression between tissue types. GTEx’s phenotype data on the donor and sample level, including histological images, offers the opportunity to probe the effect of age and sex on matrisome expression. We also incorporate data from unrelated studies on cancer, available through The Cancer Genome Atlas (TCGA), and, in a third dataset, explore idiopathic pulmonary fibrosis (IPF). In summary, we demonstrated that GTEx gene expression data reasonably correlates with protein data and can serve as normal matrisome controls for disease studies.

## Results

### Correlation of matrix gene and protein expression patterns

Prior to utilizing the matrix transcriptome for analysis, we sought to establish the extent of correlation between the transcriptome and the more typically used proteome of the matrisome. Historically, there has been concern that long-lived structural proteins would poorly correlate with their gene expression. For the purposes of this analysis we selected the core matrisome (glycoproteins, collagens, proteoglycans) and ECM regulators (n = 512 genes), and excluded the other matrisome-associated genes (ECM-affiliated proteins and secreted factors), which tend to not be integral structure elements. Two large datasets of matched proteomic and genomic (bulk RNA-sequencing) expression data exist using either label free quantification (LFQ)^8^ or a 10-plex tandem mass tag (TMT) approach^9^ to quantify protein expression across multiple tissues. The LFQ dataset used intensity-based absolute quantification (iBAQ) derived from 29 human tissues, while the TMT was from 32 GTEx tissues. The LFQ proteomic approach had better protein expression to matched gene expression correlations, highlighting challenges of normalizing TMT across samples (Supplementary Fig. S1A), and therefore we focused on the LFQ dataset for further comparisons. Despite overall poor protein/gene expression correlation in the TMT results, matrisome protein/gene correlations were generally superior to non-matrisome gene correlations (Supplementary Fig. S1B).

**Figure 1:**
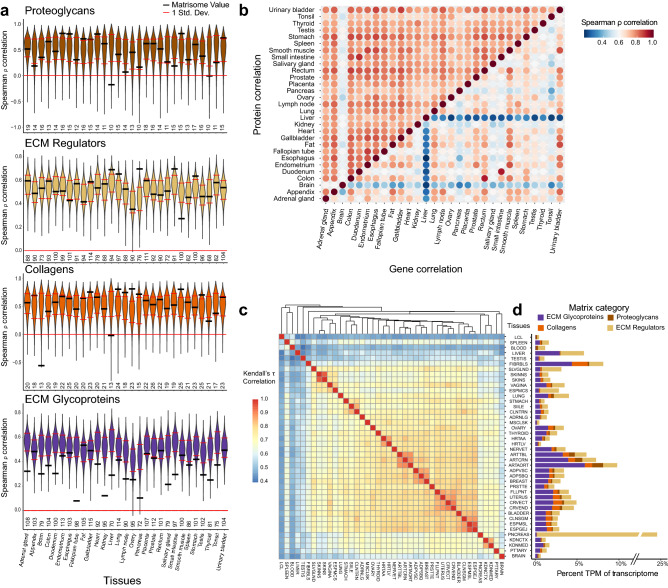
A transcriptomic and proteomic overview of the matrisome. (**a**) Four violin plots of 10,000 permutations of gene-protein Spearman’s correlations. The N of proteins per tissue is determined by how many protein/gene pairs were detected for each of the 4 matrisome classes. The black bars are the Spearman’s ρ correlation for the matrisome protein-gene pairs. (**b**) A heatmap correlation of matrisome proteins (top left) and gene mRNAs (bottom right) between tissues showing stronger correlations among proteins. (**c**) A Kendall’s τ correlation heatmap of each tissues’ median expression of matrisome genes. Multiple brain region datasets are collapsed into one tissue by using the median expression of each gene across these samples. GTEx abbreviations are used. (**d**) The median percent transcriptome of matrisome categories for each tissue. The colored bars each represent a different matrisome division.

For the 29 tissues, a matrisome correlation was generated using matrisome protein-gene pairs that had detectable levels of both the protein and transcript. Equal numbers of non-matrisome protein-gene pairs were used to generate a Spearman’s ρ distribution for each matrisome category (Fig. 1a). The matrisome Spearman’s ρ significantly correlated with the number of gene-protein pairs in each tissue (ρ = 0.773, p = 8.72e−07) indicating greater correlation with increasing data depth. For three matrisome categories, the protein-gene correlations proved as robust as any non-matrisome pairing (11 inferior, 56 equivalent and 20 superior). ECM glycoprotein protein-gene pairs underperformed comparatively in that 21 of 29 Spearman’s ρ values were inferior to non-matrix pairs (Fig. 1a, Supplementary Fig. S2). Glycoproteins are a protein class difficult to identify by general LFQ methods^10^. These findings indicate that the matrisome protein-gene correlates are equivalent to non-matrisome protein-gene correlations with transcriptomic data likely providing a superior measure of glycoproteins than general proteomic (LFQ) methods.

From the original LFQ-based manuscript, intra-tissue correlations were much stronger in the complete proteomic dataset compared with the matched transcriptomic dataset^8^. When we selected matrisome components, we observed strong similar protein inter-tissue correlations (Fig. 1b), potentially due to a short dynamic range of LFQ/iBAQ. Brain notably correlated the least with other tissues. By contrast, there was significantly more diversity in correlation across the matrisome transcripts. Pairs such as rectum and colon or small intestine (jejunum/ileum) and duodenum were strongly associated (ρ = 0.931 and 0.934 respectively), as expected. More unrelated tissues such as the brain, pancreas, and liver had expected low correlation by matrisome gene expression. The pancreas separation is likely due to the high expression of regulatory matrisome genes in comparison to other tissue types^11^, while liver has much higher levels of matrisomal glycoprotein expression^10^. These results demonstrated an overall reasonable correlation between matrix genes (transcriptomics) and proteins (proteomics) such that the evaluation of the GTEx transcriptomic dataset would prove valuable, and that the study of matrix transcriptomics allows new insight into the variation found between tissues.

### The transcriptomic matrisome clusters GTEx tissue types

Using DESeq2 normalized bulk-seq gene expression data across GTEx’s 54 tissues and 17,382 samples, we curated an overview of the matrisome’s transcriptome. Using the median of normalized tissue counts, a Kendall’s τ correlation was generated between the 54 tissue types limited to the core and regulatory matrisome (n = 512 genes) (Fig. 1c, Table S1). There was strong clustering by similar organ types (skin, adipose, artery; Min τ = 0.95, 0.87, 0.89 respectively). Overall, cell type composition is a primary driver of additional tissue clustering based on the ratio of epithelium, smooth muscle, and other cell types^12^.

We investigated genes that showed unique patterning between tissues and tissue types to better understand the inter-tissue expression that exists. Brain, for example, had enriched expression of proteoglycan stabilizers (*NCAN* and *BCAN*), while pancreas had highly-enriched expression of pancreatic enzymes *CELA3B*, *PRSS2*, and *PRSS3*. Organs containing stratified squamous epithelium, such as skin, esophagus, and vagina, shared higher expression of *SERPINA12*, *SERPINB13*, *SERPINB4*, and *LAMB4,* compared to other tissues. Glandular tissues, such as the small intestine and transverse colon also shared highly expressed matrisome genes including *MEP1A*, and *ADAMDEC1*. The liver notably had unique expression of transcripts including *ITIH2*, *SERPINC1*, *SERPINA10*, *F7*, *LPA* and *VTN*. The testis also had unique matrisome gene expression, although this may be the result of “leaky” transcription^13^. Of all sample types, the lowest global expression of matrisome genes was in EBV-transformed lymphocytes, while the liver and testis had the most uniquely expressed matrisome genes. To further understand this variation globally, we interrogated how the different matrisome sub-categories contributed to each tissue type.

We determined what percent of the transcriptome could be assigned separately to the four divisions of core matrisome and ECM regulators (Fig. 1d). Overall, similar tissue types share the same level and composition of matrisome expression. For example, three adipose-dominant tissue types (visceral adipose tissue, subcutaneous adipose tissue, and mammary breast tissue) had the same general composition and expression level of matrisome genes. The three arteries share similar compositions, with increasing matrisome transcript levels correlating with medial wall thickness^14^. EBV-transformed lymphocytes serve as a useful negative control, as they fail to generate matrix in culture. The pancreas again remains an outlier due to its high expression of pancreatic enzymes that fall into the category of ECM regulators (*PRSS1*, *PRSS2*, *CELA3A*)^11^.

### Some matrisome gene expression is modulated by age and associates with age-related histology

**Figure 2:**
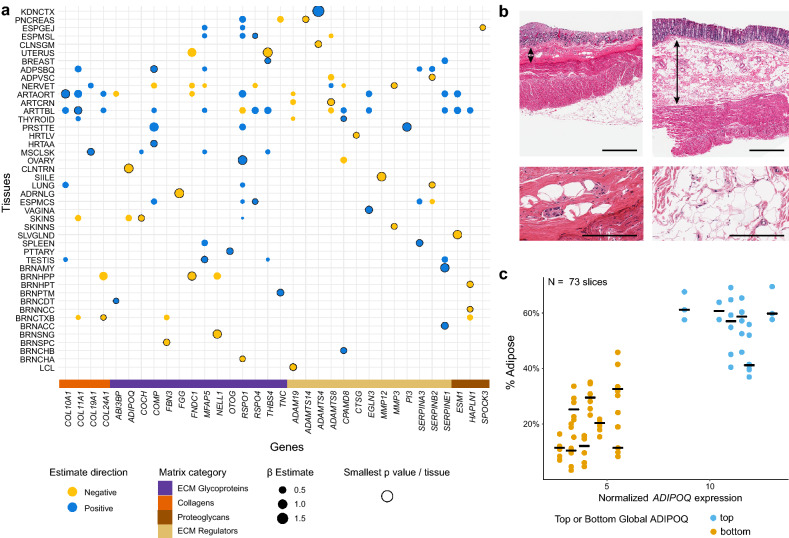
Age-associated transcriptomic changes of the matrisome. (**a**) A plot indicating the 32 transcripts with the largest change in expression relative to age. The transcript with the smallest FDR-adjusted p-value per tissue is noted along with all other instances of that transcripts having a β estimate ≥ 0.15 in other tissues. Tissues with no significant findings (FDR-adjusted p > 0.05) were not included. (**b**) Representative images of the transverse colon with varying levels of multi-tissue *ADIPOQ* expression at two different powers. The left sample, GTEX-14C5O, had low levels of *ADIPOQ* and is notable for a thin submucosa (two-headed arrow) with few adipocytes. The right sample, GTEX-1KXAM, had high levels of *ADIPOQ*, with a notable submucosal area containing large numbers of adipocytes. The bars are 1 mm and 200 µm for the low and high power views respectively. (**c**) A scatterplot representing the percent of adipose tissue in the submucosa of the transverse colon for the top 6 and bottom 8 individuals ordered on body wide *ADIPOQ* expression. The datapoints (n = 2 to 6, median of 6, per individual) of multiple measured areas per sample are colored to represent the top (blue) or bottom (yellow) multi-tissue *ADIPOQ* expressors. Bars indicate the median level of submucosal adipocytes for each individual.

Aging is a biological process often linked to diseases such as cancer, heart disease, Type II diabetes, and osteoporosis^15,16^. Aging impacts the matrisome through fibrosis and scarring, a component of which is collagen deposition. Using the limma package to regress gene expression on scaled age while correcting for sex, experimental batch, tissue ischemic time, and Hardy Scale (known batch effects^17^) we identified 1231 instances of 382 unique core and/or regulatory matrisome transcripts associating with subject age in 44 tissues (False Discovery Rate (FDR) corrected p < 0.05). Of these, 864 gene expression-tissue pairs increased with age while 367 decreased (Fig. 2a, Supplementary Fig. S3, Supplementary Table S2). The most varied transcripts by age were *MGP*, *RSPO1*, *LTBP2* and *VWCE*; these were found to be differentially expressed (DE) in 9–11 tissues each. However, none of these transcripts had the greatest effect size in any of the organs, which is why they are absent from Fig. 2a. Of the mRNAs that correlated with age, the median number of tissues they were significant in was 3. Of the 209 mRNAs that had consistent expression directionality with age across tissues, 58 decreased and 151 increased with age.

One change we noted was decreased *ADIPOQ* expression with aging in both sun exposed skin (linear model on normalized counts; β =  − 0.361, FDR-adjusted p = 5.97e−03) and transverse colon (linear model on normalized counts; β =  − 0.991, FDR-adjusted p = 3.45e−06). It is common for visceral fat to increase with age^18^, thus it was unexpected for *ADIPOQ*, a gene whose expression is associated with adipocytes, to decrease with age in these tissues. To investigate this, we subsetted out individuals ranked on multi-tissue *ADIPOQ* expression into our top (age range: 24–67 years; median: 36.5 years) and bottom (age range: 49–70 years; median: 64 years) *ADIPOQ* groups and evaluated the GTEx transverse colon digitized histologic images.

We observed that, overall, individuals with higher *ADIPOQ* levels had a thicker submucosa with more adipocytes when compared to samples from individuals with lower *ADIPOQ* levels (Fig. 2b). We quantified these differences using ImageJ to determine the percentage of inter-tissue white space, as a surrogate for adiposity of the submucosal regions. There was a significant correlation between white space and transverse colon normalized *ADIPOQ* levels in the submucosa, based on the images taken from the samples with the highest and lowest global *ADIPOQ* expression levels while controlling for BMI, sex, ischemic time, and age, with subject ID as a random effect (linear mixed model on normalized counts; β = 0.027 p = 6.75e−3, Fig. 2c, Supplementary Table S3). Age was also significant in this model (β =  − 0.005, p = 5.88e−3), indicating that the age also contributes to lower submucosal adipose levels.

## Sex differences in the matrisome are driven by sexual dimorphism

**Figure 3:**
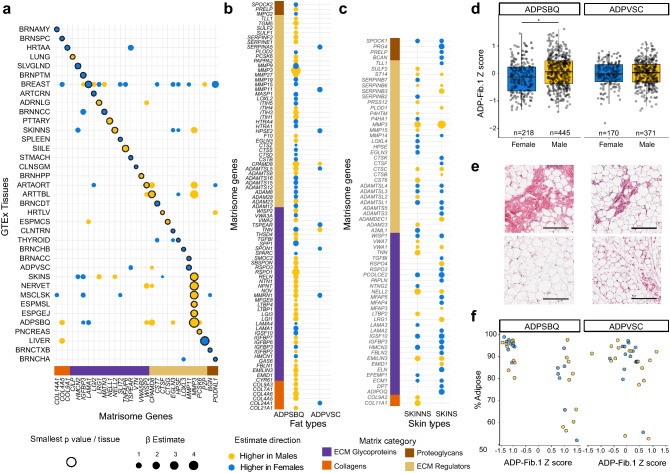
Sex based differences in the matrisome transcriptome. (**a**) A plot of sex DE genes. The transcript with the smallest p-value per tissue is noted along with all instances of that transcript having a significant β estimate in other tissues. Tissues with no significant findings were not included. (**b**) The sex-related genes that are DE in ADPSBQ and ADPVSC. Notably, ADPSBQ has 81 DE genes while ADPVSC has only 10. (**c**) The sex-related genes that are DE in skin not sun exposed (SKINNS) and skin sun exposed (SKINS). The size of the dot indicates the β estimate. The tissues are mostly congruent, having 17 shared genes with the same expression directionality. (**d**) A barplot comparing the Z score for the ADPSBQ ADP-Fib.1 gene cluster between males and females for both ADPSBQ and ADPVSC tissues. In ADPSBQ, which has more DE sex genes, cluster ADP-Fib.1 associates with the male sex. This cluster shares 11 genes with the DE gene list in (**b**). In ADPVSC, there is no sex difference. *=1.96e−05 (**e**) Adipose samples from two individuals showing differing levels of fibrosis. The top samples are from GTEX-IGN73 and the bottom samples are from GTEX-1K2DA. The left samples are ADPSBQ and the right samples are ADPVSC. The bar is 200 um. (**f**) The median percent adipose tissue from ADPSBQ (n = 34; 16F/18M) and ADPVSC n = 34; 16F/18M) samples, with the ADP-Fib.1 Z-score on the X axis. This data shows that the gene transcripts in the ADP-Fib.1 cluster positively correlate with adiposity and negatively correlate with fibrosis in ADPSBQ tissue.

Sexual dimorphism in the transcriptome is characterized by varying cell type composition^19^. Here we attempt to recapitulate these findings and explore them with a focus on matrisome changes. Tissue specific linear models, generated on matrisome gene expression with sex and age as variables identified 645 gene mRNAs DE (FDR-adjusted p < 0.05) by sex (315 unique) across 19 tissues (Supplementary Table S4). As expected, the tissue with the greatest number of DE genes was breast tissue (n = 166; Supplementary Fig. S4), which was likely driven by different cell type proportions found in males and females, specifically more adipocytes for males and more epithelial cells for females. *MMP3* was the most commonly DE gene, with significant expression differences in 9 tissues (Fig. 3a, Table S1).

Subcutaneous adipose tissue (ADPSBQ) had the second most sexually DE genes at 81. This contrasted with visceral adipose tissue (ADPVSC) only having 10 DE genes. Only one gene transcript overlapped and shared directionality between the two adipose tissues (Fig. 3b). Adipose tissue is known to be sexually dimorphic in metabolism and fat deposition^20^. Skin is also characterized by sexual dimorphism^21^, a major difference being collagen distribution^22^. Not sun exposed skin had 39 DE sex genes, while sun exposed skin had 44 DE genes (Fig. 3c). Unlike the adipose tissue samples, both skin locations shared 17 intersecting genes and expression directionality, consistent with their similar functions. Because ADPSBQ and ADPVSC have more distinct roles in the body^23^, we sought to better understand what drove differential gene expression based on sexual dimorphism.

We clustered co-correlating matrisome genes within ADPSBQ, generating 3 gene clusters. One cluster separated into two negatively correlating sub-clusters termed ADPSBQ fibrosis 1 and 2 (ADP-Fib.1, n = 33 genes; ADP-Fib.2 n = 2 genes) (Supplementary Table S5). Eleven genes overlapped between ADP-Fib.1 and the ADPSBQ linear model results, implying this cluster was sexually DE. The expression of ADP-Fib.1 was generally higher in male ADPSBQ samples (β = 0.197, p = 1.96e−05) as determined by a linear model, controlled for BMI on the normalized gene expression from the ADP-Fib.1 cluster (mean = 0, sd = 0.587). This same relationship was not seen in ADPVSC (Fig. 3d, Supplementary Table S6).

To better understand this incongruency, we examined the GTEx ADPSBQ histology samples most associated with the extreme ends of the ADP-Fib.1 cluster normalized score, quantifying the amount of adiposity and the inverse amount of fibrosis. This was performed in 73 ADPSBQ and 66 ADPVSC tissue samples from both males (n = 36) and females (n = 32) at the extreme ends of the subcutaneous ADP-Fib.1 Z-scores (Fig. 3e). There was a significant association between the ADP-Fib.1 cluster Z-score and adipose percentage (linear model; p = 2.56e−03) in a regression model correcting for sex, BMI, and ischemic time, in ADPSBQ tissue (Fig. 3f). Again, there was no association with ADPVSC (linear model; p = 0.07) using the same model.

### Carcinomas from different tissues have similar matrisome genes changes

**Figure 4:**
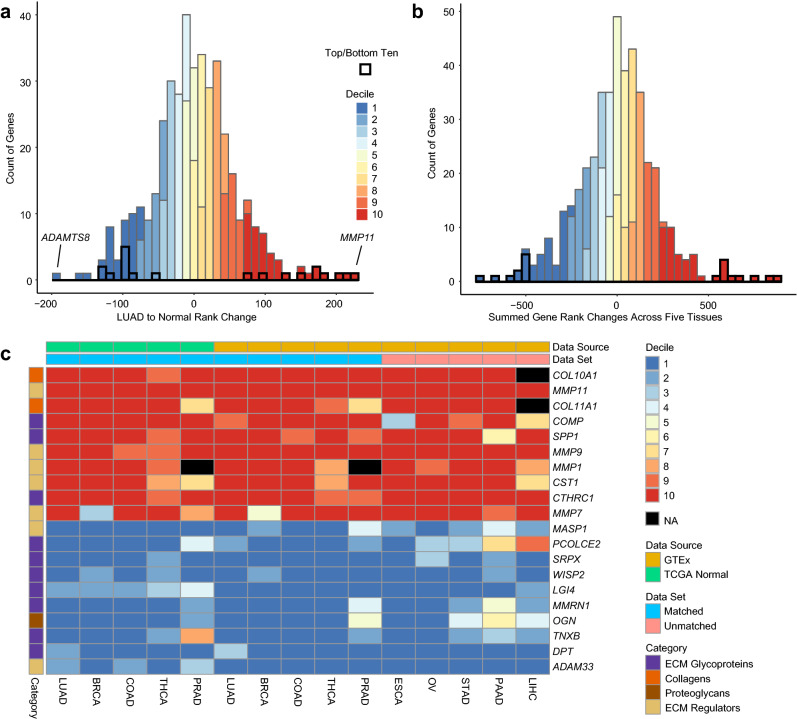
Commonly upregulated and downregulated matrisome gene transcripts. (**a**) A histogram of transcript rank changes between combined TCGA normal samples (n = 110) and GTEx samples (n = 374) compared to TCGA cancer samples (n = 542) in lung. The histogram is colored on the decile of rank change. (**b**) A histogram of the sum of normal to cancer rank changes for gene transcripts across lung, breast, colon thyroid and prostate (n = 443). The histogram is colored on the decile of rank change. (**c**) A heatmap of the top and bottom rank-changed matrisome gene transcripts between normal tissue and cancer. The rank change order is split into deciles, (1—1st decile to 10—10th decile). NAs are represented by black boxes and indicate no expression in the normal or cancer samples.

We then explored the usefulness of GTEx-based matrisome as a control dataset for cancer stroma studies. To do this, we acquired and compared GTEx and TCGA RNA-seq data for lung adenocarcinoma, breast invasive carcinoma, colon adenocarcinoma, thyroid carcinoma, and prostate adenocarcinoma normal tissues and cancers (LUAD, BRCA, COAD, THCA, PRAD, Supplementary Table S1)^24^. We used the DESeq2 variance stabilizing transformation separately on each tissue and their respective datasets (GTEx, TCGA-normal, or TCGA-cancer) generating appropriate normalized gene expression used to evaluate the relative rank change of expression between paired normal (TCGA or GTEx) and cancer samples. The relative rank order method mitigated the expected batch effects problem of working across these datasets. This approach allowed us to identify gene transcripts that increased in cancer such as *MMP11* (increase rank by 223 positions) and gene transcripts with lower expression in cancer such as *ADAMTS8* (decrease rank by 196 positions) (Fig. 4a).

By summing these normal to cancer rank changes across tissues we generated a list of the top 10 matrisome gene transcripts most consistently in the top or bottom decile of rank changes, revealing common tissue agnostic cancer expression changes (Fig. 4b, Supplementary Table S7). These gene transcripts were detected equally well between tumor samples matched to TCGA normal (38 of 50 in the 10th decile; 33 of 50 in the 1st decile) or GTEx paired organ samples (39 of 50 in the 10th decile; 41 of 50 in the 1st decile) and represent a fairly consistent set of cancer-associated matrisome changes. We then extended this experiment to esophageal carcinoma, ovarian serous cystadenocarcinoma, stomach adenocarcinoma, pancreatic adenocarcinoma, and liver hepatocellular carcinoma (ESCA, OV, STAD, PAAD, LIHC, Supplementary Table S1) using only paired GTEx normal tissues. In this group, the overexpressed matrisome gene transcripts remained consistently in the upper decile (40 of 50), while the lower expressed transcripts trended in the same direction but were not as consistently in the bottom decile (29 of 50, Fig. 4c).

We confirmed these common matrisome alterations, in six unrelated microarray expression datasets of paired lung, breast, esophagus, and colon carcinomas and normal tissues (Supplementary Table S8). Differential expression analysis on these datasets demonstrated an average of 8.5 of 10 10th decile genes and 7.8 of 10 1st decile genes were significantly DE in the correct direction across these samples.

We noted elevated *COL10A1* expression is associated with a type of cancer associated fibroblast (CAF) and further evaluated the relationship of the cancer associated matrisome to fibroblast type^25^. We obtained a pan-cancer (ovarian, lung, colorectal) single cell RNA-sequencing dataset, to evaluate CAFs and non-CAF fibroblasts (myofibroblast, ovarian stroma cell, fibroblast like excitable cell, etc.; abbreviated as FIB)^26^. A Pearson correlation matrix on the 20 genes from our bulk-sequencing analysis showed clustering based on their rank change profile (increase or decrease, Supplementary Fig. S5a). These genes were then evaluated for expression differences between fibroblast types. A Wilcoxon Rank Sum Test identified all 20 genes were significantly variable between CAFs and FIBs. All rank increase genes were higher in CAFs while all but one rank decrease gene (*MMRN1*) was higher in FIBs (Supplementary Fig. S5b, Supplementary Table S9).

These findings indicate that GTEx tissue matrisome transcript signatures can be a robust control tissue for tumor studies, that a fairly consistent set of up or downregulated matrisome genes are altered among numerous malignancies, and these changes are likely related to the presence of CAFs.

### The matrisome can distinguish idiopathic pulmonary fibrosis from normal and acutely injured lung

Idiopathic pulmonary fibrosis (IPF) is a chronic disease with a progressive decrease in lung function due to increased fibrosis and ECM remodeling^27,28^. We were interested in determining if matrisome gene expression data could correlate with the known histopathologic change of IPF and determine if GTEx matrisome data could be used as a control tissue for disease studies. For this, we identified a published gene expression dataset of IPF^29^ and integrated this data with the lung GTEx expression data.

We performed clustering analysis of a bulk sequencing IPF dataset (Sivakumar et al.^29^) containing 46 IPF samples, 8 acute lung injury (ALI) samples and 26 control lung samples along with 20 GTEx samples that we previously indicated were normal or had ventilator-associated injury^12^, focusing on the core matrisome genes and ECM regulators^29^. We identified one expression cluster that split into two negatively correlating sub clusters, LUNG-Fib.1 and LUNG-Fib.2 (Supplementary Fig. S6, Supplementary Table S10). A normalized expression Z-score of gene transcripts in each cluster was used to test associations of clusters LUNG-Fib.1 and LUNG-Fib.2 to disease. A linear model, controlling for batch, age, sex, and tobacco use indicated that both ALI and IPF were significantly associated with the cluster LUNG-Fib.1 expression Z-score, with IPF having a larger effect size (linear model; p = 0.017, 3.71e-19; β = 0.47, 1.63; for ALI and IPF respectively; Supplementary Table S11). Cluster LUNG-Fib.2 comparatively only correlated with IPF (linear model; p = 0.0001; β =  − 0.778).

**Figure 5:**
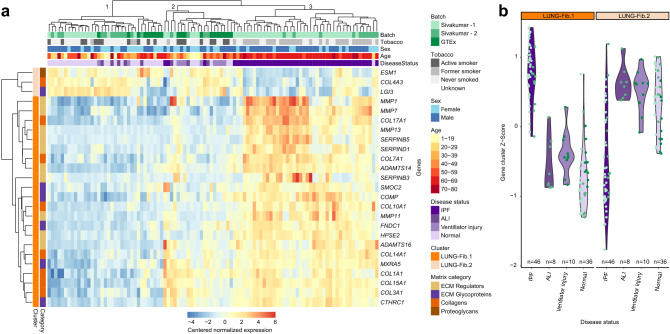
Idiopathic Lung Fibrosis characterization using GTEx. (**a**) A heat map representing the centered normalized expression of matrisome genes in clusters LUNG-Fib.1 or LUNG-Fib.2 between lung samples. The x-axis of the lung samples indicates disease, batch, tobacco usage, sex, and age status of each. Age and being in batch Sivakumar—2 significantly correlate with IPF samples (logistic regression; p = 0.012; p = 0.029 respectively). (**b**) A violin-sina plot of the normalized score of gene expression cluster LUNG-Fib.1 and LUNG-Fib.2 comparing IPF, acute lung injury, ventilator injury, and normal lung samples from Sivakumar et al*.* The color of each data point indicates the batch.

Hierarchical clustering on the lung samples limited to the gene transcripts from clusters LUNG-Fib.1 and LUNG-Fib.2, identified three sample groupings (Fig. 5a). The first grouping contained 35 samples of normal lung, ALI, and ventilator injury from both the GTEx and Sivakumar datasets (Table 1). It was marked by low expression of gene cluster LUNG-Fib.1 and higher expression of gene cluster LUNG-Fib.2. The second group of 24 samples was a mix of normal, ventilator injury, ALI, and IPF samples, with its expression profile appearing to be an intermediate between groups 1 and 3 for the cluster LUNG-Fib.1 and cluster LUNG-Fib.2. The third grouping contained 40 IPF samples and one normal sample. Clear differences of the LUNG-Fib.1 and LUNG-Fib.2 gene expression cluster Z-scores can be seen for IPF samples relative to the other injury or non-injury patterns of lung (Fig. 5b). Taken together, this data indicates both that GTEx matrisome data can be usefully integrated with other gene expression datasets and that the matrisome itself can distinguish disease states.

**Table 1:** Lung sample clusters related to disease.

	Cluster 1	Cluster 2	Cluster 3	Disease sum
Normal lung	7	28	1	36
Ventilator injury	8	2	0	10
Acute lung injury	3	5	0	8
Idiopathic pulmonary fibrosis	6	0	40	46
Sample sum	25	35	41	

## Discussion

Using the GTEx dataset, we have curated a body-wide understanding of the transcriptomic matrisome in health and limited disease states. The large dataset of GTEx, and others like it, may improve our collective understanding of the matrisome by providing complementary data to proteomic and functional studies. As tissue transcriptomic studies are more easily performed and generate deeper datasets than proteomic-based methods, establishing a correlation between the two types of data was critical.

We initially validated gene expression as a marker of proteomic levels through the Wang et al*.* LFQ proteomic/gene expression study, and found good correlation with the exception of glycoproteins^8^. However, this poor correlation likely reflects the challenge of capturing glycoproteins by proteomics rather than a weakness of the transcriptomics, as transcriptomic/proteomic correlations were consistently robust across the other three matrisome classes^10^. Overall, matrisome transcriptomic data more appropriately clustered similar tissues based on their cell type composition, such as epithelial tissues or smooth muscle tissues, than proteomics, which showed a more uniform and inappropriate correlation pattern (Fig. 1c).

The second proteomic study, 10-plex TMT, was performed on GTEx tissues and was expected to correlate well with the transcriptomic data^8^. It did not perform as well. One of the challenges of a 10-plex TMT when working with diverse samples, is having a control sample on each TMT that contains every peptide needed to normalize proteins for all tissues. The TMT approach pooled reference samples across 32 organs, which diluted out rare/organ-specific proteins (~ 1/32nd of the peptide mix) reducing capture of these peptides and decreasing the organ specificity of control peptides.

While other studies have investigated aging throughout all GTEx gene expression on earlier versions of GTEx data, we focused on just matrisome gene expression aging correlations^30^. Melé et al*.* generated a list of 47 DE matrisome genes due to aging across all GTEx tissues. With a larger dataset, we identified 382 significantly changed matrisome genes analyzed individually across multiple tissues, capturing all but three of the genes in their dataset (*MMP23B, IGFBPL1,* and *LEPRE1*). However, of the 44 shared genes, only 73% shared global directionality. These discrepancies are likely due to their analysis being on all genes, resulting in a stricter p-value threshold as well as not including similar batch effects or their usage of tissues as a random effect in their model.

Our findings reveal that there are tissue specific age related changes in the matrisome. Some have bodywide directionality such as a decrease in *LGI3* in 6 tissues*,* and an increase in *MPG* in 11 tissues, the former being associated with adipokines and cytokines^31^, the latter being a known protein in the DNA repair pathway^32^. Other gene transcripts did not always share body-wide directionality such as *LOXL2* and *SLIT2* which implies genomic tissue-specific matrisome changes with age. Using GTEx’s histology data we were able to elucidate an association between *ADIPOQ*, a gene transcript decreasing with age in two tissues, and loss of fat in the transverse colon sub-mucosal space. Our findings are inconsistent with other literature stating that inter-mucosal fat increases with BMI^33^ and does not decrease with age^34^. However our data differs from theirs in several regards. Wada et al*.* studied the fat using liquid droplets, and Mesa et al*.* looked at submucosal thickness as a measure of fat in comparison to our approach which evaluated the percentage of submucosal space by histology.

Our analysis has also generated a list of matrisome specific differences between the sexes. Breast tissue, due to its sexual dimorphism, was logically the tissue with the most differentially expressed genes. The most prevalent sex differentially expressed gene was *MMP3*. Previous work has shown that the MMP3 protein is elevated in male serum and plasma when compared to females^35,36^. However, our finding appears to be the first description of *MMP3* differences across multiple tissues and at the gene level. The fat and skin data had striking differences between tissues and sex (Fig. 3c,d). ADPSBQ had 82 DE genes, while ADPVSC had 10 DE genes, and of the four shared transcripts, only *CPAMD8* shared directionality. ADPSBQ DE genes were primarily higher in males (63%) while ADPVSC was higher in females (80%). Conversely, skin sun exposed and skin not sun exposed with 44 and 40 DE genes respectively shared a larger proportion of transcripts, 17 total all sharing directionality, with no strong difference in sex association (65% higher in females and 50% higher in females respectively). There are important functional differences between ADPSBQ and ADPVSC characterized by sex-specific and hormone-based differences^23,37^, while the skin sampling locations (suprapubic and lower leg) were expected to be more similar. We identified a gene cluster (ADP-Fib.1, Table S5) in adipose tissue that was increased in fibrosis and among males. It included generic fibrosis markers such as *COL1A1* and fat specific fibrosis markers like *COL6A6*, which is known to inhibit adipocyte growth and result in an increased inflammatory profile^38^. This finding nicely connected transcriptomic data to the histologic and phenotypic data available in GTEx. Sex DE matrisome genes were investigated previously using GTEx v6^19^. That study found 294 unique sex DE matrisome genes, on a per tissue basis, of which 195 overlapped with our findings. Differences likely result from methodological approaches, GTEx versions, and multiple testing corrections.

Integrating GTEx and TCGA allowed us to explore the cancer matrisome. Focusing on carcinomas, a combination of GTEx and TCGA tissues identified 20 matrix genes with consistent high or low expression across tumor types. The top gene, *COL10A1* is a well-known marker of solid tumors^39^ and *COL11A1*, the third highest gene, is a marker of cancer-associated fibroblasts^40^. A previous pan-cancer single cell analysis of ovarian, lung, and colorectal cancer, showed that *COMP*, *COL10A1, COL11A1*, and *MMP1* all have higher expression in CAFs^26^. Our own analysis of that dataset showed that 19 of 20 matrix genes correlated appropriately with CAFs or non-CAFs. These rank changes, validated in unrelated gene microarray datsets, and by fibroblast single cell data, show the robustness and rationale of this gene set.

Using the high variance matrisome gene clusters, we were also able to parse out associated DE genes that may play roles in the pathology of IPF. A prior manuscript evaluated global gene mRNA changes in IPF describing *COL1A1* and *MMP7* changes in IPF, similar to our findings, along with several gene transcripts (*LAMB3*, *LAMC2*, *FN1*, and *TNC*) not uncovered in our project^28^. However, Tsukui et al. showed in IPF that *COL1A1* + fibroblasts co-express *CTHRC1* and *COMP*, both of which were also found in our IPF associated cluster^41,42^. The mix of collagens (7 genes) and matrix metalloproteinases (4 genes) also implies ECM remodeling occurring in the fibrotic lung^43^. Importantly, the GTEx samples overlapped appropriately with the non-IPF samples of the Sivakumar study, indicating their value as a control tissue source for other matrisome studies across a range of disease states.

Across our analyses multiple genes appeared repeatedly, raising questions to their biological importance. (Supplemental Table S12). Twelve matrisome genes were variable across four studied categories (*COL10A1, COL11A1, COMP, CTHRC1, FNDC1, LGI3, MASP1, MMP7, MMP11, MMRN1, MXRA5,* and *OGN*). *LGI3* was normally associated with lower fibrosis and was decreased in both skin samples with age. This is consistent with prior analyses showing a relationship between *LGI3* and melanocytes, a cell type that decreases in density with age^44,45^. *LGI3* was also found in the the LUNG-Fib.2 cluster, which was lower in IPF. *LGI3* is associated with type II pneumocytes, a cell type that is reduced due to increased apoptotic activity in IPF^46^. Separately, *COL1A1*, a known marker of fibrosis, increased in two tissues (tibial artery and the anterior cingulate cortex) and decreased in one (pancreas) relative to subject age. It was also increased in adipose and IPF fibrosis clusters, consistent with its fibrosis association.

This study has several limitations. First, GTEx data is skewed, with a bias for male subjects (n = 653 males, n = 327 females) and a bias for older individuals (mean = 52.76 years, median = 55 years, standard deviation = 12.91 years). The lack of younger individuals likely impacts the aging analysis of matrix genes that reach steady-state later in life^47^. Contamination from highly expressed genes in GTEx, is known, and could impact on specific expression patterns^11^. There were also limitations due to GTEx’s available histology. Not all individuals had histology samples of the tissues we interrogated, and for some, the tissue orientation precluded image analysis. While it would have been interesting to evaluate the tumor matrisome in metastases, TCGA had too few samples of this type.

In conclusion, patterns of matrisome gene expression approximate the measured values of matrix proteins. As a result, the GTEx database is a robust resource for transcriptomic matrisome gene expression to explore matrix genes variability across tissues, age and sex. Further, in two examples, it demonstrated usefulness as a control tissue source for disease studies and can be used in this capacity to augment further investigations into the human matrisome.

## Materials and methods

### Retrieval and correlation of label free quantification/genomic datasets

We acquired protein and protein-coding gene data^8^ found in their Supplementary Tables S1 and S2 respectively. The proteins were analyzed by mass spectrometry using identification and intensity based absolute quantification (iBAQ)^48^. Methods of RNA extraction of the tissues, including library preparation and sequencing are described in Uhlén et al*.*^49^. Our analysis used their fragments per kilobase of transcript per million mapped reads (FPKM) data.

All analyses were completed in R v.4.0.2 unless stated otherwise. For each tissue, we linked their protein iBAQ and gene FPKM data together. The data was filtered down to matrisome core and regulatory protein-gene pairs that had non-0 values in their gene and protein columns. Using base R, Spearman’s rank correlations were generated for the following comparisons: correlations between gene transcripts and proteins within each tissue; and a distribution of correlations for each tissue, randomly sampling N non-0 protein-gene pairs (without replacement) 10,000 times using dplyr 1.0.2. The N of samples is the number of viable matrisome gene transcripts for each tissue. This process was repeated for each matrisome category.

### Retrieval and correlation of GTEx proteomic-transcriptomic data

Protein data and gene data were retrieved from Supplementary Tables S2D and S3A from Jiang et al.^9^. The protein data used was “Cleaned relative protein abundances in log2 scale” and the gene data was “Cleaned and normalized RNA log2 (TPM) expression for all protein-coding genes across matched samples”. We also acquired their gene across tissue correlation data from Supplemental Table S4A.

From the proteomic and transcriptomic data, samples from individuals with proteomic and transcriptomic data were selected for the analysis. The protein and gene samples were joined and filtered, requiring the protein-gene pairs to have non-0 and non-NA values in each column. We then generated a Spearman’s rank correlation for each sample pair on all gene transcripts, and the core and regulatory matrisome.

Using the body wide gene tissue correlations from Jiang et al*.* (Supplemental Table S4A; 1-s2.0-S0092867420310783-mmc5.xlsx, sheet 2) each protein-gene pair correlation was labeled with its matrisome category or as “Non-Matrisome”. Each matrisome category’s correlations were compared against the correlation of all other genes using a Mann–Whitney U test. The resulting p-values were corrected for multiple tests using Benjamini–Hochberg method, and the data was presented using ggplot2 (v.3.3.2).

### Retrieval and processing of GTEx bulk sequencing and phenotype data

The gene read counts of the RNA-Seq GTEx version 8 data set (GTEx_Analysis_2017-06-05_v8_RNASeQCv1.1.9_gene_reads.gct.gz) were downloaded from the GTEx Portal (https://gtexportal.org/home/datasets), along with the de-identified sample and subject annotations (GTEx_Analysis_v8_Annotations_SampleAttributesDS.txt, GTEx_Analysis_v8_Annotations_SubjectPhenotypesDS.txt). GTEx v8 median TPM for each tissue was downloaded through the GTEx portal (GTEx_Analysis_2017-06-05_v8_RNASeQCv1.1.9_gene_median_tpm.gct.gz). Numeric ages of the GTEx subjects were acquired through dbGap with approval. Pertaining to human data, all methods were carried out in accordance with relevant guidelines and regulations.

For the median tissue expression, raw read counts were normalized together using the VST (variance stabilizing transformation) feature in DESeq2 (v.1.22.1) in R version (v.3.6.117). This method incorporates estimated size factors based on the median-ratio method, and transformed by the dispersion-mean relationship.

The median VST normalized read counts for core matrisome and ECM regulator genes were used to develop a tissue median expression profile. The median expression of all separate brain tissues were used to form the median BRAIN tissue. A clustered heatmap of tissues were generated using a Kendall’s τ correlation matrix. The heatmap was plotted using Pheatmap (v.1.0.12) and the tissues were clustered using a 1 − Kendall’s τ correlation matrix based on their Euclidian distance. Matrisome genes were labelled with their respective categories, and each category was separately summed for each tissue, and then divided by the total TPMs within each tissue. These values were plotted using ggplot2.

For the age and sex analysis raw read counts, by tissue type, were filtered using the filterByExpr function from edgeR (v.3.32.1)^50^ before being analyzed using the voom method^51^ in limma (v.3.46.0)^52^ in R version (v.4.0.2). GTEx tissues were filtered to only core matrisome and ECM regulator genes. Independently, each of these genes were modeled as a linear function of scaled age (using R’s base scale function) and sex corrected for ischemic time (SMTSISCH), Hardy Scale (DTHHRDY) and experimental batch (SMGEBTCH) using limma’s lmFit function. The results for all tissues were collated together and had a FDR correction applied to their p-values. Only estimates with a > 0.05 corrected p-value were used for further analysis. The data was plotted using ggplot2, limited to genes with smallest p-values for the given covariate in each tissue.

### Sex based differences of adipose tissue and transverse colon

For both ADPSQB and ADPVSC, previously VST normalized genes were required to have a mean normalized read count > 5 and an across sample variance > 1.5 for inclusion in the cluster analysis. The filtered genes were clustered using hierarchal clustering on a distance generated by 1 − Kendall’s rank-correlation coefficient. A τ critical value was calculated based on the number of samples and genes expressed. The correlation-based dendrogram was cut to produce gene clusters with an average cluster correlations of at least the τ critical value.

Normalized expression scores were calculated by subtracting the mean expression and dividing by the median absolute deviation of the expression values for each gene across all samples within a given tissue. The equation is as follows, where *x* is the VST normalized expression of gene *j* in sample *i*, *t(i)* is the tissue type for sample *i*, and *J* is the number of genes in a given cluster**.**

$${S}_{i}= \frac{1}{J}\sum_{j=1}^{J}\frac{{x}_{ij} - {\overline{x} }_{j}}{\begin{array}{c}Median\\ m:t\left(m\right)=t\left(i\right)|{x}_{mj}-{\tilde{x }}_{j}|\end{array}}.$$

Using the previously generated ADP-Fib.1 cluster score, 5 males and 5 females were selected from the highest and the lowest ADP-Fib.1 scores for both ADPSBQ and ADPVSC. These samples were then de-identified and randomized to prevent biased ImageJ scoring.

Previously VST normalized transverse colon, sigmoid colon, ADPSBQ, and ADPVSC samples were selected on the requirement that all four tissues were available for a given individual. This RNA-seq data was limited to only the *ADIPOQ* gene, and normalized reads were averaged across the tissues generating the multi-tissue *ADIPOQ* score. The individuals were sorted on the multi-tissue *ADIPOQ*, and the top 6 individuals and the bottom 8 individuals were selected to have their transverse colon analyzed using ImageJ.

The percent adipose content of each sample was determined using ImageJ 2.0.0 (FIJI)^53^. For the transverse colon, only samples with submucosal space present were evaluated. The ImageJ freehand selection tool, captured the area of the submucosal space, excluding veins, white space, and muscle. For ADPSBQ and ADPVSC, the fat area was captured, avoiding white space and vasculature. The images were transformed to greyscale, a threshold was applied (range 199 to 255 light) and the percent of captured area being either adipose (white space) or fibrosis (colored space) was calculated.

Two linear models were applied on both ADPSQB and ADPVSC. The first model used BMI and sex as covariates for the ADP-Fib.1 score. The second model used ADP-Fib.1 score, sex, and BMI as covariates for percent adipose of the tissue samples. Individuals with more than one sample per tissue had the average of the percent adipose tissue used as their value.

For the transverse colon model, multi-tissue normalized *ADIPOQ* score, age, and BMI were used as covariates on percent adipose tissue in transverse colon samples. As there were multiple tissues slices per person, subject ID was used as a random effect in the model. The R package lme4 (v.1.1-26) was used to generate the linear mixed model. In the model equation (below), β_1_ is the *ADIPOQ* Score for sample *i*, β_2_ is the age of individual *j*, β_3_ is the sex of individual *j*, β_4_ is the BMI of individual *j*, *u*_*n(i)*_ is the random effect of the individual for sample *i*, and ϵ_i_ is the error term for said sample.

$${Y}_{ij}= {\beta }_{0}+ {\beta }_{1}{ADIPOQ Score}_{i}+ {\beta }_{2}{Age}_{j}+ {\beta }_{3}{Sex}_{j}+ {\beta }_{4}{BMI}_{j}+{u}_{n(i)}+ {\epsilon }_{i}.$$

### Generating tissue-agnostic rank changes between normal and cancer samples

GTEx data and TCGA data was acquired through the R package TCGAbiolinks v.2.18.0. The normal GTEx and normal TCGA tissues downloaded were colon (n = 376 GTEx [transverse colon], n = 51 TCGA), prostate (n = 119 GTEx, n = 52 TCGA), breast (n = 92 GTEx [female], n = 111 TCGA), thyroid (n = 361 GTEx, n = 59 TCGA), lung (n = 374 GTEx, n = 110 TCGA), ovary (n = 108 GTEx), stomach (n = 204 GTEx), pancreas (n = 197 GTEx), esophagus (n = 331 GTEx [ESPMCS]), and liver (n = 136 GTEx), with their respective TCGA cancers being COAD, PRAD, BRCA, THCA, LUAD, ESCA, OV, STAD, PAAD, LIHC. Using TCGA histological types, COAD was limited to colon adenocarcinoma (n = 426), prostate to prostate adenocarcinoma acinar type (n = 489), breast to infiltrating ductal carcinoma (n = 788), thyroid to thyroid papillary carcinoma (classical) (n = 357), ovary to serous cystadenocarcinoma (n = 430), stomach to both stomach adenocarcinoma and stomach intestinal adenocarcinoma (n = 306), pancreas to pancreas-adenocarcinoma ductal type (n = 147), and liver to hepatocellular carcinoma (n = 356). Lung adenocarcinoma (n = 542) and esophageal carcinoma (n = 184) did not require further filtration. Normal TCGA samples for esophagus, ovary, stomach, liver, and pancreas were excluded.

Separately, all tissues and their datasets, GTEx normal (GTEx-N), TCGA normal (TCGA-N), and TCGA cancer (TCGA-C) were filtered down to genes with an across sample sum > 5 raw counts (N_min_/N_max_ = 49,463–56,114 genes, liver and stomach respectively) and normalized using the DESeq2 VST function. After normalization, genes were further filtered to include only those with a sample mean > 5 normalized counts (N_min_/N_max_ = 20,881–25,075 genes, liver and thyroid respectively) generating a total gene whitelist for the analysis. This allowed inclusion of genes that were lowly expressed in normal tissue, but had higher expression in cancer tissue.

The above analysis was replicated after normalization steps and whitelist formation, filtering down to core matrisome and ECM regulatory genes (N_min_/N_max_ = 353–410, liver and lung respectively). GTEx-N, TCGA-N, and TCGA-C datasets were combined together and quantile normalized using the package preprocessCore (v.1.52.0) (https://github.com/bmbolstad/preprocessCore), before being separated again. The quantile normalized mean counts for genes in each dataset were calculated. An average was taken between the GTEx-N’s and TCGA-N’s mean quantile normalized counts to form a joined normal mean (Joined-N; Supplementary Fig. S7). Each gene list was ordered on their mean expression, generating for each tissue a list of ranked matrisome genes from GTEx-N, TCGA-N, TCGA-C, and Joined-N used to calculate a numerical rank change for each gene, between normal and cancer tissues (Joined-N compared to TCGA-C, Supplementary Fig. S8). For esophagus, ovary, stomach, liver, and pancreas, GTEx-N was used in place of Joined-N.

Relative rank changes of genes between TCGA-C and Joined-N were determined for lung, breast, colon, thyroid, and prostate tissue. This was replicated between TCGA-C and GTEx-N for esophagus, ovary, stomach, liver, and pancreas. The TCGA-C to Joined-N rank changes per gene were summed across the five tissues to establish a multi-tissue normal-to-cancer rank change. This highlighted genes that consistently increased or decreased across cancer samples, while lowering the effect of tissue specific changes or changes inadvertently caused by the rank change of other genes. The rank changes were segmented into deciles and the top and bottom 10 genes from the multi-tissue rank change were subsetted out and plotted using Pheatmap.

Six separate microarray studies were used to recapitulate the matrisome cancer findings. Two lung datasets (GSE31210^54,55^, GSE19188^56^), two breast datasets (GSE15852^57^, GSE109169^58^), an esophagus dataset (GSE161533 [https://www.ncbi.nlm.nih.gov/geo/query/acc.cgi?acc=GSE161533]), and a colon dataset (GSE44076^59^) were obtained through GEOquery 2.58.0. DE genes were discovered using the limma 3.46.0 R package’s eBayes function^52^ with Holm’s adjustment for p-value correction. For each gene, if ≥ 50% of probes for a given gene were called as significantly DE, the gene was considered DE.

Single-cell phenotype and normalized expression fibroblast data was downloaded from http://scope.lambrechtslab.org/ (visited on 9/22/2021), from their “Pan-cancer blueprint of tumor microenvironment” study^26^. The downloaded data was limited to the 20 analyzed rank change genes (*COL10A1, MMP11, COL11A1, COMP, SPP1, MMP9, MMPO1, CST1, CTHRC1, MMP7, MASP1, PCOLCE2, SRPX, WISP2, LGI4, MMRN1, OGN, TNXB, DPT*, and *ADAM33*). In R, all cells marked as “Low quality” were removed from the dataset before the cells were split into CAF cells and non-CAF cells (FIB) based on their “Phenotype” column. The gene correlation heatmap was created using R’s base correlation function and pheatmap function, clustering the data on the Euclidian distance of 1 − Pearson correlations. The gene’s normalized values were compared between groups using a Wilcoxon Rank Sum Test, and multiple testing was accounted for by using the Holm method.

### Acquisition, processing, and gene variance clustering of IPF data

From GEO, the design, gene annotations, and raw counts for GSE134692 were downloaded, including their IPF, ALI, and normal samples (n = 46, 8, and 26 respectively)^29^. The data was processed as the adipose tissue above, however the variance filter was a > 2 variance in this analysis. This returned two sub-clusters of high variance matrisome genes for the IPF dataset.

Histological images of GTEx lung samples were previously categorized as normal or ventilator injured by a lung pathologist^12^. From this group, 10 samples were randomly selected from both the ventilator injury samples and the normal samples.

GTEx lung samples and samples from Sivakumar et al. were combined into one DESeqDataSet the design including both disease status (IPF, ALI, Ventilator Injury, and normal) and Batch (Sivakumar-1, Sivakumar-2, and GTEx). Sivakumar et al. states they correct for these batches, but do not give the process further description. The data was normalized using DESeq2’s VST transformation. After transformation, the data was filtered to only contain the high variance IPF gene clusters (n = 25) before being centered on the average gene expression across samples. The samples and genes were clustered using Euclidian distancing and graphed using the Pheatmap package.

## Supplementary Information

Supplementary Figures.

Supplementary Tables.

## Data Availability

The dataset of proteomic (LFQ) and genomic correlations analyzed in this study (Fig. 1, Supplementary Figs. S1, S2) is published in Wang et al.^8^ publication in their Supplementary Tables S1 and S2. https://www.ncbi.nlm.nih.gov/pmc/articles/PMC6379049/bin/MSB-15-e8503-s003.zip, https://www.ncbi.nlm.nih.gov/pmc/articles/PMC6379049/bin/MSB-15-e8503-s004.zip. The dataset of proteomic (TMM) and genomic correlations analyzed in this study (Supplementary Fig. S1) is published in Jian et al.^9^ in their Supplementary Tables S2D and S3A. https://ars.els-cdn.com/content/image/1-s2.0-S0092867420310783-mmc2.xlsx, https://ars.els-cdn.com/content/image/1-s2.0-S0092867420310783-mmc3.xlsx. The GTEx dataset analyzed in Figs. 1, 2, 3, 4 and 5 including RNA-seq, sample, and histology data were acquired through the GTEx portal^60^ while subject data was acquired with special permissions through dbGAP. https://storage.googleapis.com/gtex_analysis_v8/rna_seq_data/GTEx_Analysis_2017-06-05_v8_RNASeQCv1.1.9_gene_reads.gct.gz, https://storage.googleapis.com/gtex_analysis_v8/annotations/GTEx_Analysis_v8_Annotations_SampleAttributesDS.txt. The datasets analyzed in Supplementary Table S7 were published in Yamauchi et al., Hou et al., Pau et al., Chang et al., and Solé et al. (GEO accession numbers: GSE19188, GSE31210, GSE15852, GSE109169, GSE44076, respectively). Only one dataset, curated by Qiu et al., did not have an associated paper (GEO accession number: GSE161533). The datasets analyzed in Fig. 5 and Supplementary Fig. S6 were published in Sivakumar et al. (GEO accession number: GSE134692). The TCGA dataset^24^ analyzed in Fig. 4 was attained from the Genomic Data Commons harmonized database through the R package TCGAbiolinks v.2.18.0, implementation of this code can be found in the GitHub repository under the file: quantile_rank_analysis_all_tissue.R. All sequencing data used in this study is previously publicly available. All analysis method information and code has been deposited at GitHub https://github.com/tnieuwe/Matrisome_GTEx_analysis.
