# Supplementary material for: Tissue, age, sex, and disease patterns of matrisome expression in GTEx transcriptome data
Source: Sci Rep. 2021 Nov 3;11:21549. doi: 10.1038/s41598-021-00943-x (PMC8566510; doi:10.1038/s41598-021-00943-x)
Supplement: Supplementary file 1 — Supplementary Figures. [file 41598_2021_943_MOESM1_ESM.pdf]

Tissue, age, sex, and disease patterns of matrisome expression in GTEx transcriptome data

Tim O. Nieuwenhuis[1], Avi Z. Rosenberg[1], Matthew N. McCall[2], Marc K. Halushka[1]*

1 - Department of Pathology, Johns Hopkins University School of Medicine, Baltimore, MD, 21205 USA
2 - Department of Biostatistics and Computational Biology, University of Rochester Medical Center, Rochester, NY 14642, USA

Additional file 1: Supplemental Figures S1-S8

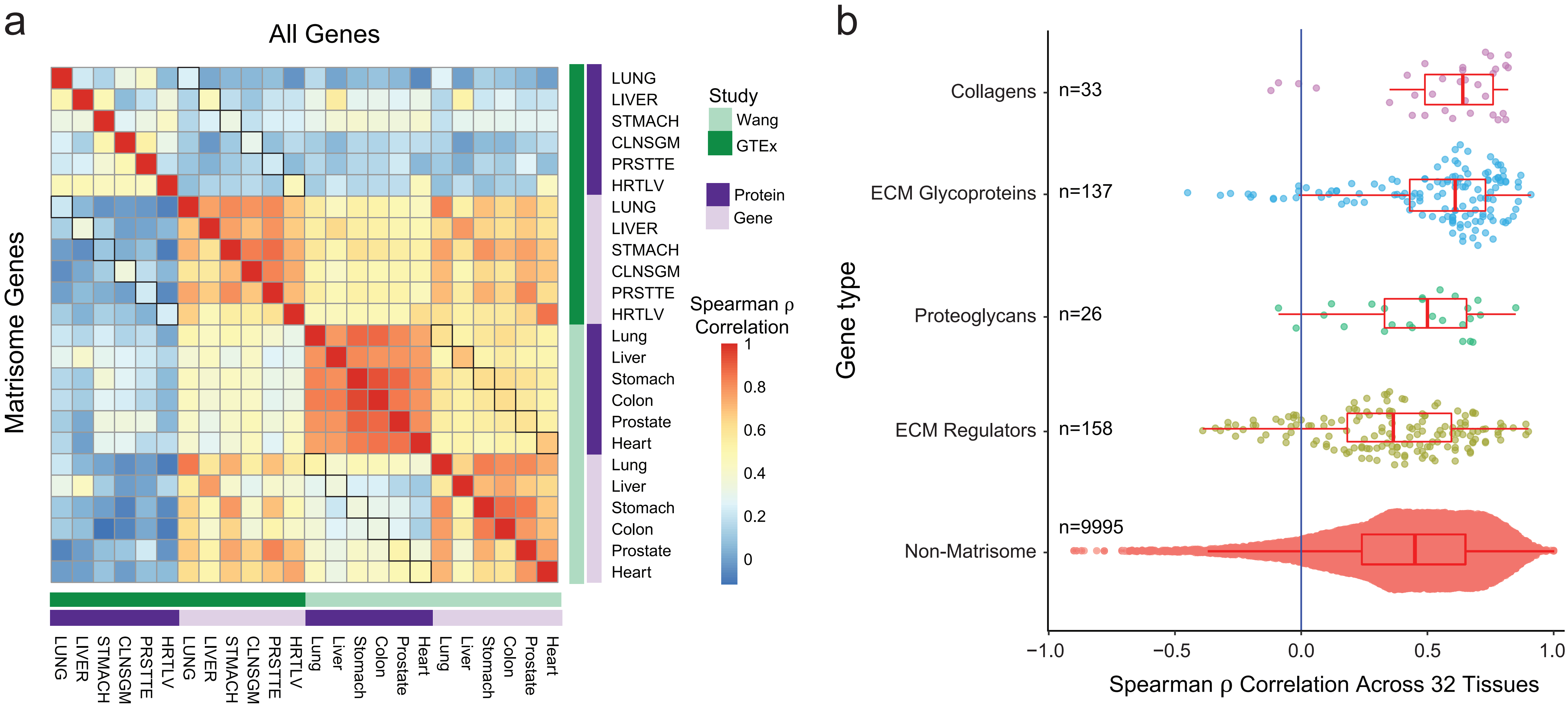

a
All Genes
Matrisome Genes
LUNG
LIVER
STMACH
CLNSGM
PRSTTE
HRTLV
LUNG
LIVER
STMACH
CLNSGM
PRSTTE
HRTLV
Lung
Liver
Stomach
Colon
Prostate
Heart
Lung
Liver
Stomach
Colon
Prostate
Heart
Study
Wang
GTEx
Protein
Gene
Spearman ρ Correlation
1
0.8
0.6
0.4
0.2
0
b
Gene type
Collagens
n=33
ECM Glycoproteins
n=137
Proteoglycans
n=26
ECM Regulators
n=158
Non-Matrisome
n=9995
−1.0
−0.5
0.0
0.5
1.0
Spearman ρ Correlation Across 32 Tissues

Figure S1. GTEx proteomic data (TMT) poorly correlates with GTEx transcriptome data while Wang *et al.* proteomic data (LFQ) correlates well with both GTEx and Wang transcriptomic data.

(a) A Spearman's correlation heatmap of six tissues shared across GTEx and Wang *et al.* studies between proteomic and genomic datasets, all genes on the top portion and matrisome genes on the bottom. Using randomly selected representative samples of GTEx tissues, we correlated the shared genes between the two datasets for each tissue (all genes min = -0.193 max = 0.914, matrisome genes min = -0.258, max = 0.942). Black box outlines indicate key correlations. Overall, the GTEx proteomic data (TMT) correlates the worst with the other datasets. (b) A sina and boxplot of different matrisome categories correlations across tissues in the GTEx proteomic (TMT) and genomic dataset. Collagens and glycoproteins significantly outperformed non-matrisome genes, while ECM Regulators underperformed (Mann-Whitney; p = 2.71e-03, 1.32e-06, and 6.03e-03 respectively). Proteoglycans did not significantly over or under perform non-matrisome genes.

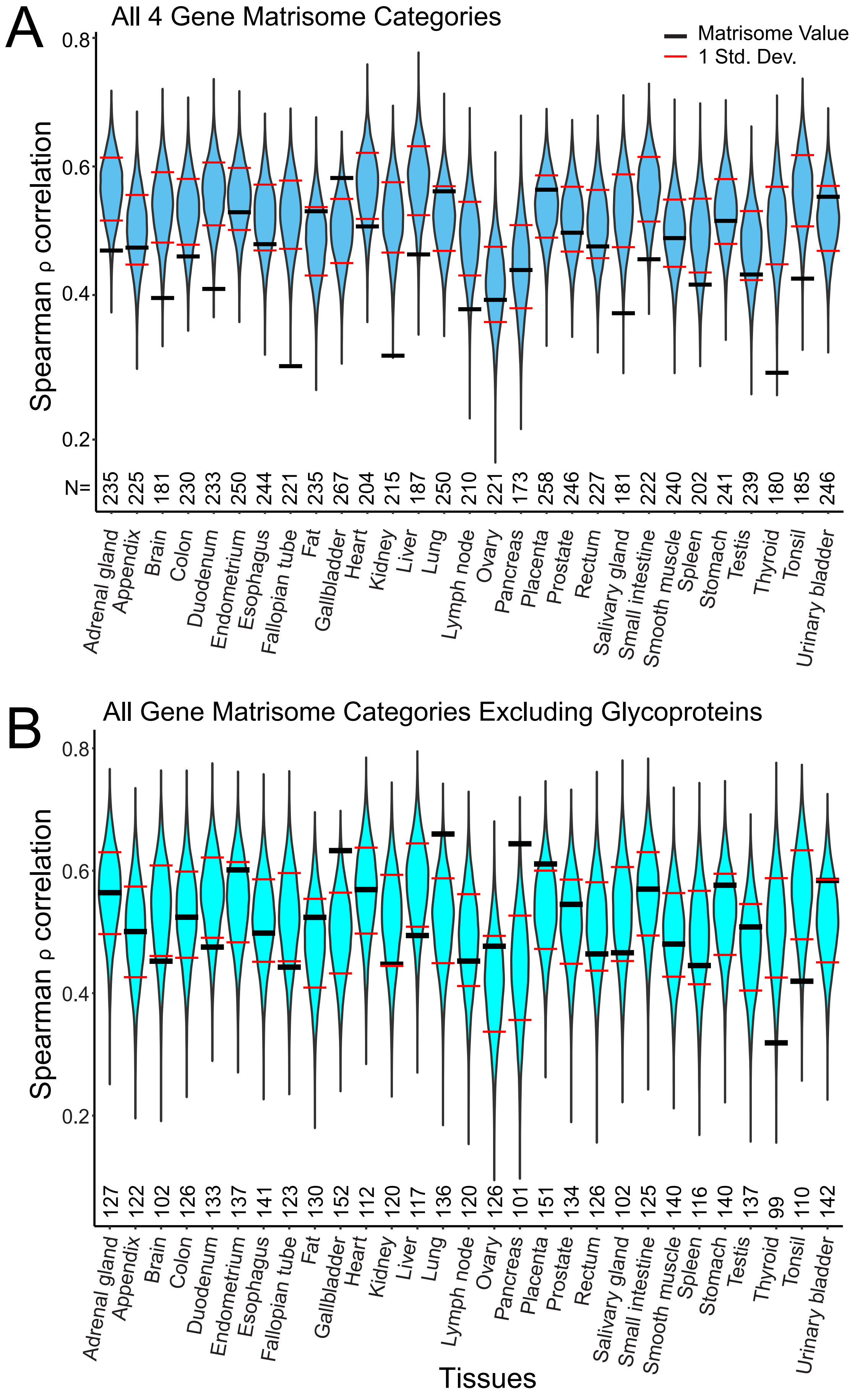
A
All 4 Gene Matrisome Categories
Matrisome Value
1 Std. Dev.
Spearman ρ correlation
0.8
0.6
0.4
0.2
N=
235 225 181 230 233 250 244 221 235 267 204 215 187 250 210 221 173 258 246 227 181 222 240 202 241 239 180 185 246
Adrenal gland
Appendix
Brain
Colon
Duodenum
Endometrium
Esophagus
Fallopian tube
Fat
Gallbladder
Heart
Kidney
Liver
Lung
Lymph node
Ovary
Pancreas
Placenta
Prostate
Rectum
Salivary gland
Small intestine
Smooth muscle
Spleen
Stomach
Testis
Thyroid
Tonsil
Urinary bladder
B
All Gene Matrisome Categories Excluding Glycoproteins
Spearman ρ correlation
0.8
0.6
0.4
0.2
127 122 102 126 133 137 141 123 130 152 112 120 117 136 120 126 101 151 134 126 102 125 140 116 140 137 99 110 142
Adrenal gland
Appendix
Brain
Colon
Duodenum
Endometrium
Esophagus
Fallopian tube
Fat
Gallbladder
Heart
Kidney
Liver
Lung
Lymph node
Ovary
Pancreas
Placenta
Prostate
Rectum
Salivary gland
Small intestine
Smooth muscle
Spleen
Stomach
Testis
Thyroid
Tonsil
Urinary bladder
Tissues

Figure S2. Protein-transcript pair violin plots of 10,000 sampled Spearman's correlations and each tissue's matrisome correlation.

The matrisome underperforms sampled distributions with the inclusion of glycoproteins, but performs well with their exclusion.

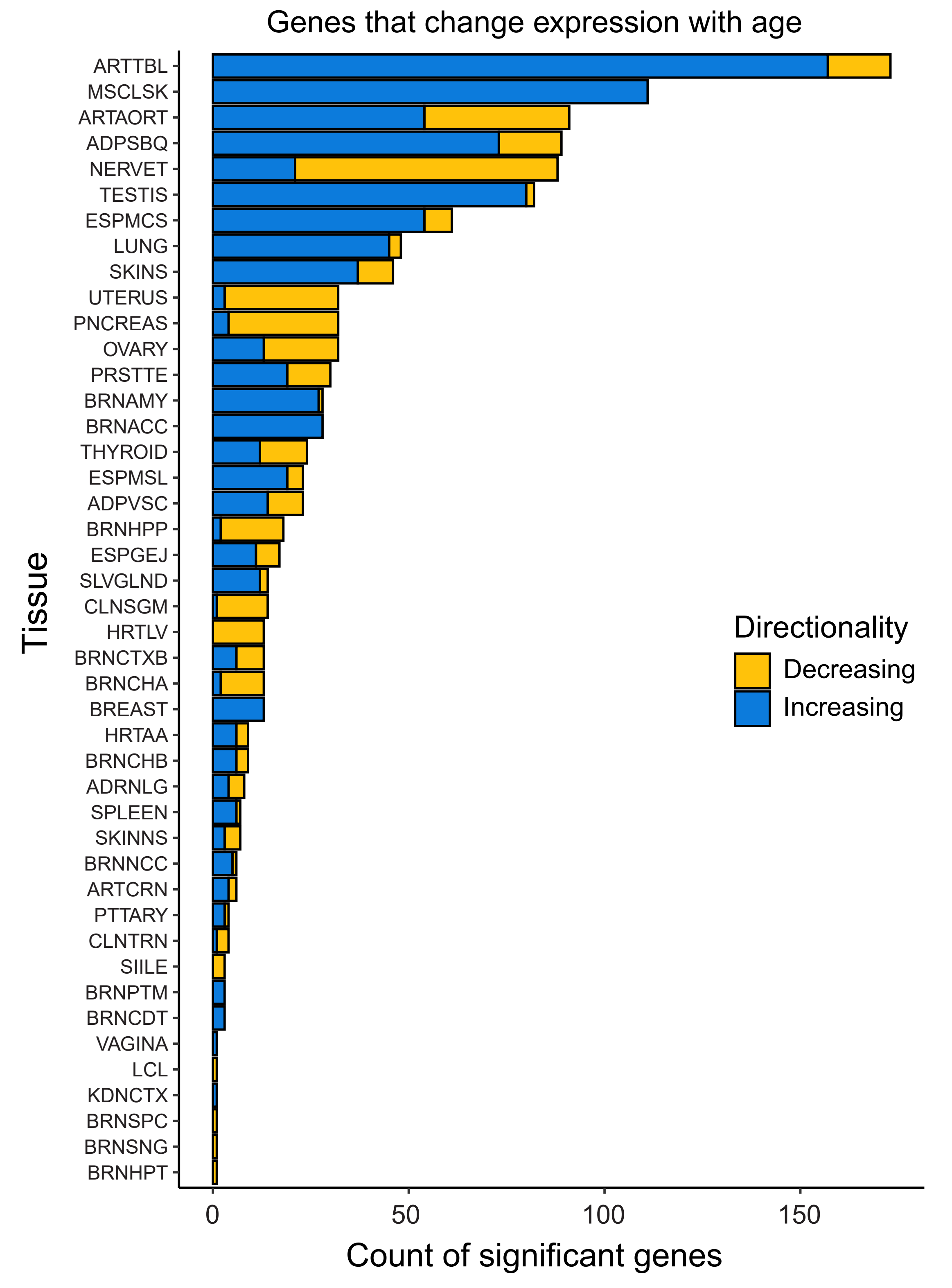
Genes that change expression with age
Tissue
ARTTBL
MSCLSK
ARTAORT
ADPSBQ
NERVET
TESTIS
ESPMCS
LUNG
SKINS
UTERUS
PNCREAS
OVARY
PRSTTE
BRNAMY
BRNACC
THYROID
ESPMSL
ADPVSC
BRNHPP
ESPGEJ
SLVGLND
CLNSGM
HRTLV
BRNCTXB
BRNCHA
BREAST
HRTAA
BRNCHB
ADRNLG
SPLEEN
SKINNS
BRNNCC
ARTCRN
PTTARY
CLNTRN
SIILE
BRNPTM
BRNCDT
VAGINA
LCL
KDNCTX
BRNSPC
BRNSNG
BRNHPT
0
50
100
150
Count of significant genes
Directionality
Decreasing
Increasing

Figure S3. A barplot count of matrisome genes for each GTEx tissue that significantly change with age based on a linear model controlling for sex. Blue represents genes that positively correlate with age while yellow presents genes that negatively correlate with age.

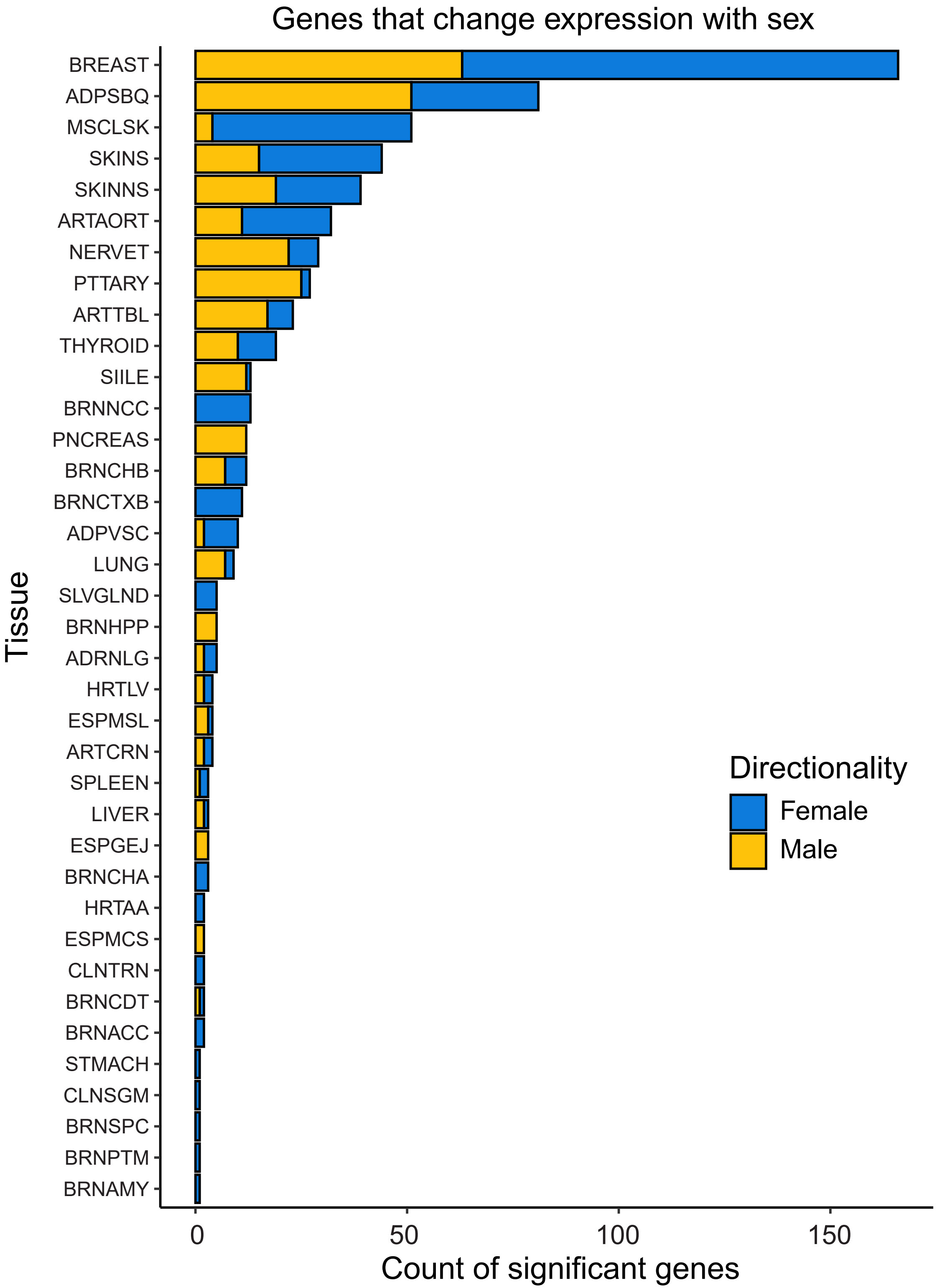
Genes that change expression with sex
Tissue
BREAST
ADPSBQ
MSCLSK
SKINS
SKINNS
ARTAORT
NERVET
PTTARY
ARTTBL
THYROID
SIILE
BRNNCC
PNCREAS
BRNCHB
BRNCTXB
ADPVSC
LUNG
SLVGLND
BRNHPP
ADRNLG
HRTLV
ESPMSL
ARTCRN
SPLEEN
LIVER
ESPGEJ
BRNCHA
HRTAA
ESPMCS
CLNTRN
BRNCDT
BRNACC
STMACH
CLNSGM
BRNSPC
BRNPTM
BRNAMY
0
50
100
150
Count of significant genes
Directionality
Female
Male

Figure S4. A barplot count of matrisome genes for each GTEx tissue that significantly change with sex based on a linear model controlling for age. Blue represents genes that are associated with higher expression in females while yellow presents genes that have higher expression in males.

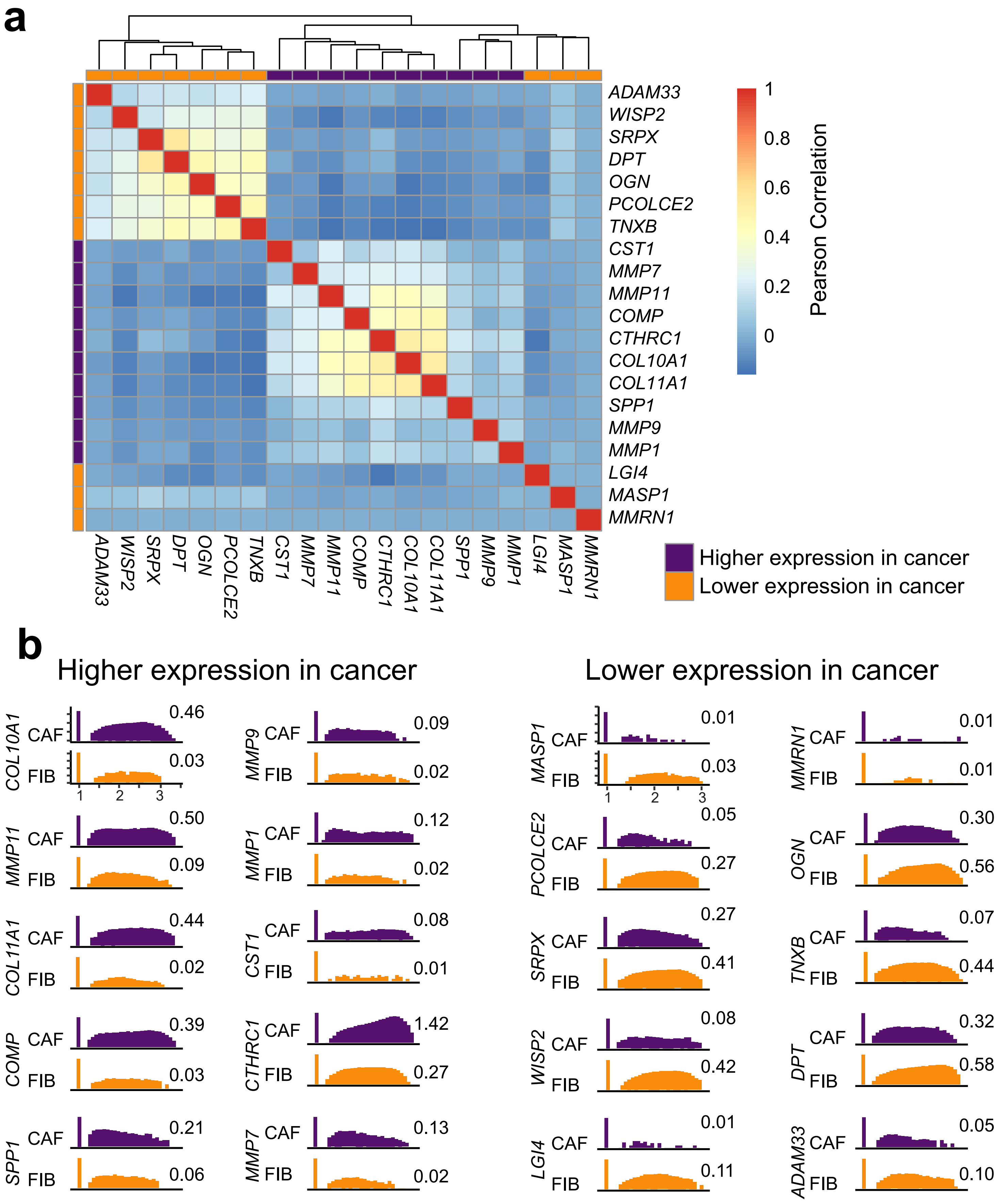

a
ADAM33
WISP2
SRPX
DPT
OGN
PCOLCE2
TNXB
CST1
MMP7
MMP11
COMP
CTHRC1
COL10A1
COL11A1
SPP1
MMP9
MMP1
LGI4
MASP1
MMRN1
Pearson Correlation
1
0.8
0.6
0.4
0.2
0
Higher expression in cancer
Lower expression in cancer
b
Higher expression in cancer
Lower expression in cancer
CAF
FIB
COL10A1 0.46 0.03
MMP11 0.50 0.09
COL11A1 0.44 0.02
COMP 0.39 0.03
SPP1 0.21 0.06
MMP9 0.09 0.02
MMP1 0.12 0.02
CST1 0.08 0.01
CTHRC1 1.42 0.27
MMP7 0.13 0.02
MASP1 0.01 0.03
PCOLCE2 0.05 0.27
SRPX 0.27 0.41
WISP2 0.08 0.42
LGI4 0.01 0.11
MMRN1 0.01 0.01
OGN 0.30 0.56
TNXB 0.07 0.44
DPT 0.32 0.58
ADAM33 0.05 0.10
1
2
3

Figure S5. Single-cell data recapitulates bulk sequencing normal to cancer findings in cancer associated fibroblasts. (**a**) High and low expressed cancer matrisome genes in a Pearson correlation matrix from single cell data (N genes = 20, N cells = 23,513). The genes are clustered on the Euclidian distance of their correlations. (**b**) Histograms of normalized expression values + 1 for genes in either CAFs (N = 6,978) or non-cancer associated fibroblasts (FIB) (N = 16,535). The Y-axis is log10-scaled individually for each histogram, while the X-axis is fixed within a single gene group to the max value between the CAF and FIB cells and divided into 30 equal bins. The numbers to the upper right of each plot is the mean normalized values for the group.

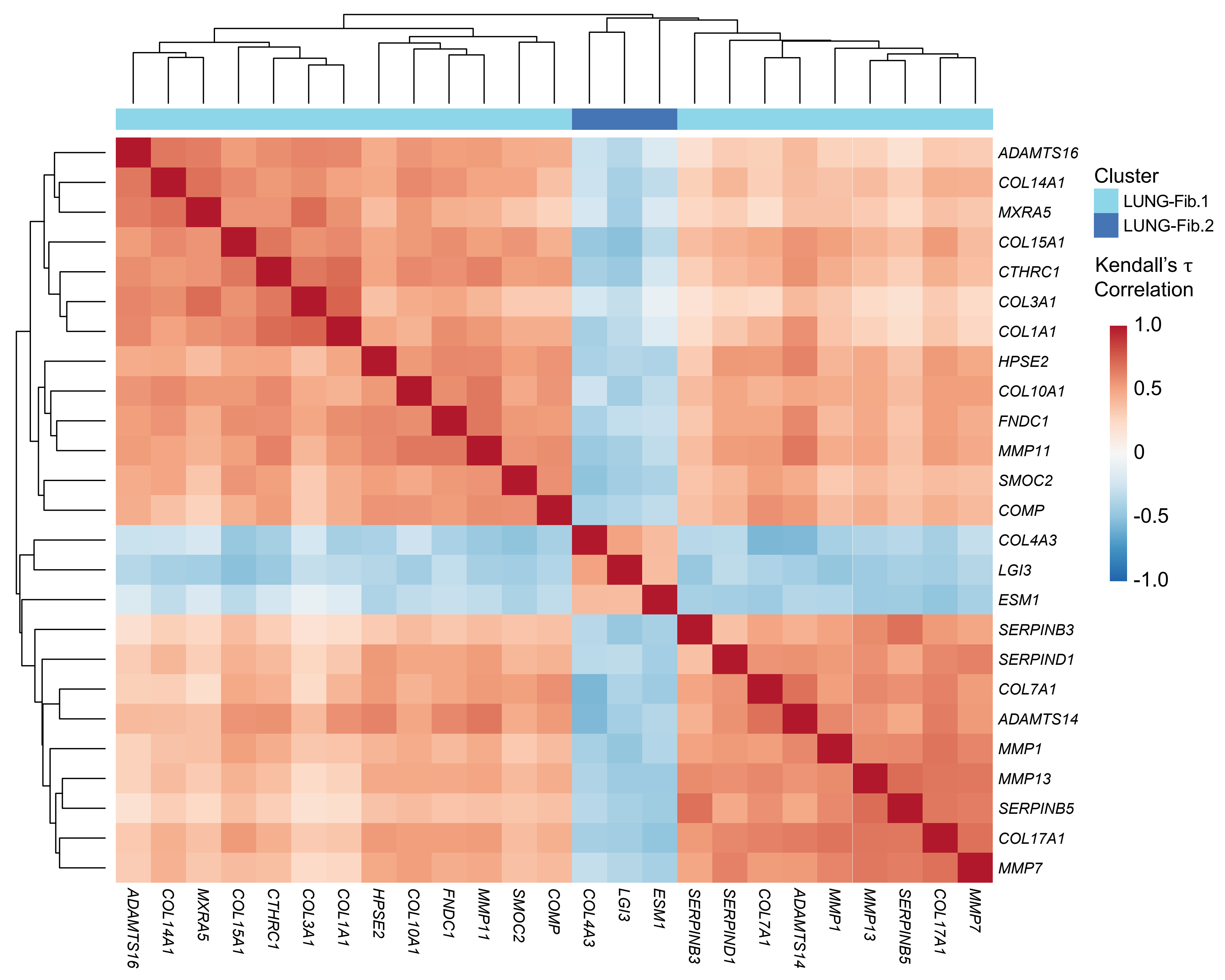
Cluster
LUNG-Fib.1
LUNG-Fib.2
Kendall's τ Correlation
1.0
0.5
0
-0.5
-1.0
ADAMTS16
COL14A1
MXRA5
COL15A1
CTHRC1
COL3A1
COL1A1
HPSE2
COL10A1
FNDC1
MMP11
SMOC2
COMP
COL4A3
LGI3
ESM1
SERPINB3
SERPIND1
COL7A1
ADAMTS14
MMP1
MMP13
SERPINB5
COL17A1
MMP7

Figure S6: A heat map of the Kendall's τ correlation matrix between high variance matrisome genes in the GSE134692 data set that includes IPF, ALI, and normal lung tissue. The light blue cluster represents LUNG-Fib.1 a cluster that associates with IPF samples while LUNG-Fib.2 represents gene transcripts the correlate with ALI, ventilator injury, and normal lung.

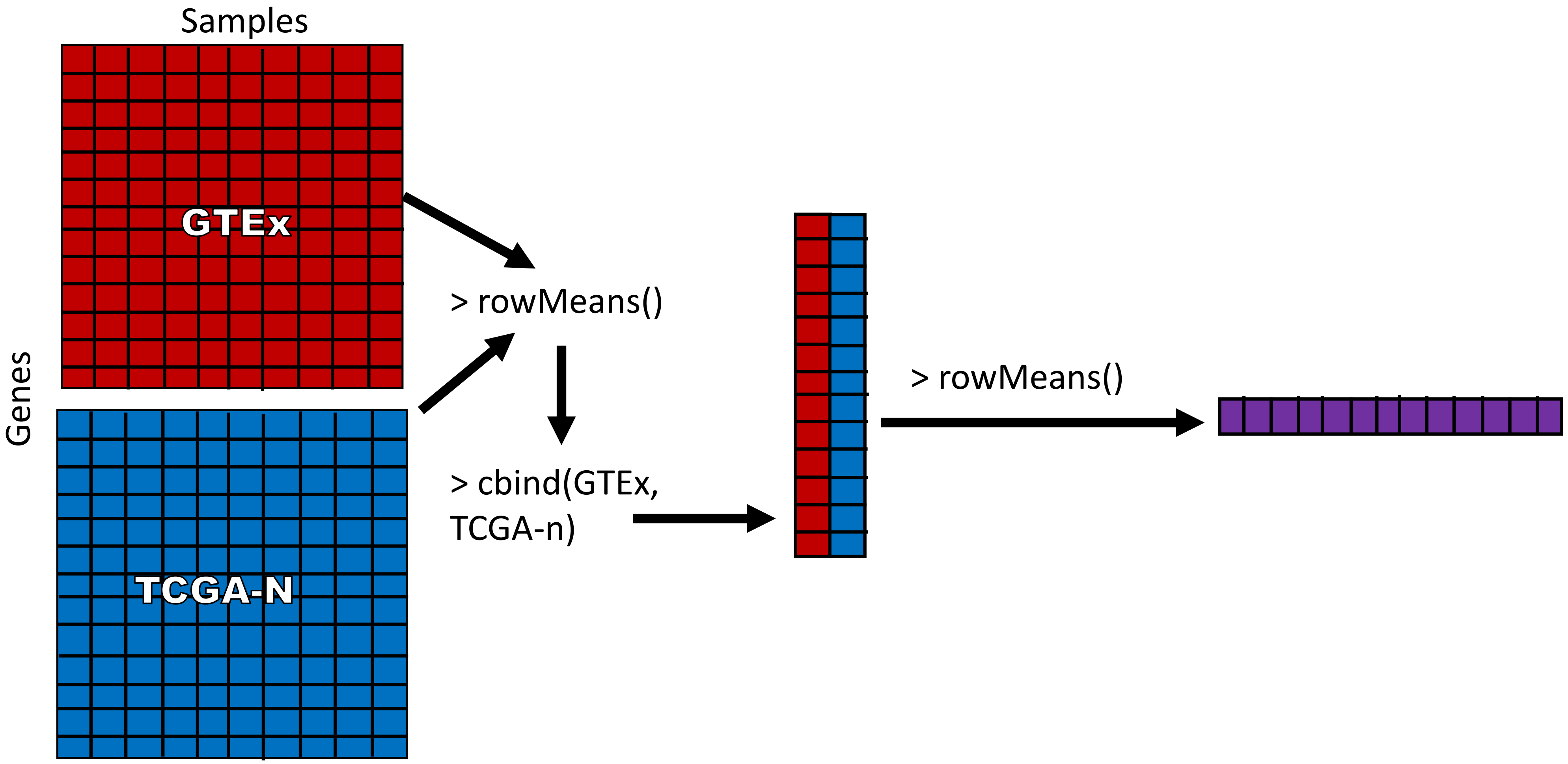
Samples
Genes
GTEx
TCGA-N
> rowMeans()
> cbind(GTEx, TCGA-n)
> rowMeans()

Figure S7: Visual illustration of generating the Joined-N gene list using R code. The red matrix represents normalized GTEx-N data while the blue matrix represents normalized TCGA-N data. The joined vectors are the appropriate row means, and the purple vector is the means between GTEx-N and TCGA-N vectors for each gene.

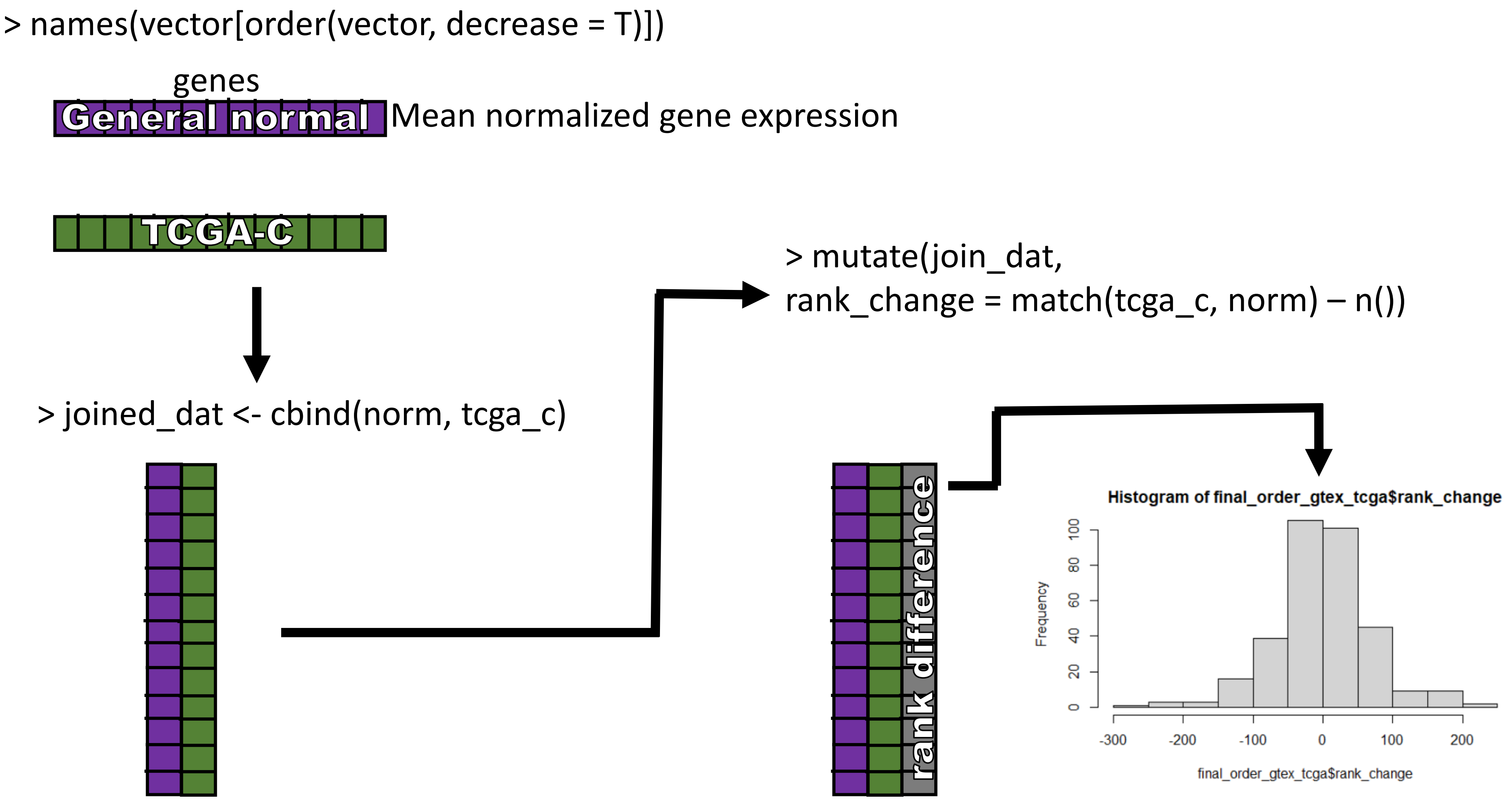

> names(vector[order(vector, decrease = T)])
genes
General normal
Mean normalized gene expression
TCGA-C
> joined_dat <- cbind(norm, tcga_c)
> mutate(join_dat,
rank_change = match(tcga_c, norm) – n())
rank difference
Histogram of final_order_gtex_tcga$rank_change
Frequency
0
20
40
60
80
100
-300
-200
-100
0
100
200
final_order_gtex_tcga$rank_change

Figure S8: Visual illustration of generating normal to cancer rank change using R code. Joined-N data, represented in purple, was ordered on the mean values and joined to the green ordered TCGA-C data. Using the Dplyr mutate function a new column of rank differences was calculated, represented in grey.
